# Complete Genome of *Bacillus megaterium* Siphophage Staley

**DOI:** 10.1128/genomeA.00864-13

**Published:** 2013-12-05

**Authors:** Waylon J. Hastings, Matthew A. Ritter, Karthik R. Chamakura, Gabriel F. Kuty Everett

**Affiliations:** Center for Phage Technology, Texas A&M University, College Station, Texas, USA

## Abstract

Siphophage Staley was isolated because of its ability to grow on *Bacillus megaterium*. Here we report the complete genome and annotation of phage Staley and describe core features. Among its interesting genes is one encoding an SleB germination protein.

## GENOME ANNOUNCEMENT

*Bacillus megaterium* is an aerobic, spore-forming bacterium found in diverse biological environments (1). *B. megaterium* is useful in producing medicinal proteins such as penicillin amidase and glucose dehydrogenase for penicillin production and blood glucose tests, respectively (2). Bacteriophages of *B. megaterium* are of particular importance as they offer the possibility of biotechnological exploitation.

Bacteriophage Staley was isolated from a soil sample collected in College Station, TX. Phage DNA was sequenced using 454 pyrosequencing at the Emory GRA Genome Center (Emory University, Atlanta, GA). Trimmed FLX Titanium reads were assembled to a single contig at 17.05-fold coverage using the Newbler assembler, version 2.5.3 (454 Life Sciences), at default settings. Contigs were confirmed to be complete by PCR. Genes were predicted using GeneMarkS (1) and corrected using software tools available on the Center for Phage Technology (CPT) Portal (https://cpt.tamu.edu/cpt-software/portal/). Electron microscopy was performed at the Microscopy and Imaging Center at Texas A&M University.

Phage Staley has a host range that includes *B. megaterium* Km Sp^−^, QM B1551, PV361, and WH320 strains. Staley has an 81,089-bp double-stranded DNA (dsDNA) genome, a GC content of 35.7%, and a coding density of 90.7%.

Several genes encoding phage replication/recombination proteins were identified, including those encoding DNA polymerase III epsilon subunit (exonuclease), helicase, primase, RecB family exonuclease, and a RuvC-like Holliday junction resolvase. DNA biosynthesis proteins were identified (ribonucleotide reductase alpha and beta subunits, guanylate kinase, thymidylate synthase, and dUTPase). Few structural proteins were detected by BLASTp and InterPro Scan analysis (2, 3). Structural proteins identified were a tail fiber protein, a tape measure protein with a lysin domain, and a tail spike protein with a pectin lyase domain. It is hypothesized that the pectin lyase domain is involved in exopolysaccharide depolymerization to allow phage diffusion through biofilm (4). A large terminase subunit was identified, although it showed no homology to TerLs from phages of known packaging strategies; thus, a packaging strategy cannot be inferred. Based on sequence similarity to *B. megaterium* phage Slash (KF669661), the terminal repeat is presumed to be 567 bp. Phage Staley carries a holin and two lysin genes. The holin is a class II holin with two transmembrane domains in an N-in C-in topology. Of the two lysin candidates, the SleB-like *N*-acetylmuramyl-l-alanine amidase has a predicted N-terminal signal peptidase I cleavage site. A cytoplasmic l-alanyl-d-glutamate peptidase was found adjacent to the holin.

Finally, Staley has genes consistent with a temperate lifestyle. The phage encodes an Rha superfamily protein (IPR014054) with an Ant1-like C-terminal domain (IPR005039). In enterobacterial phage P1, the C-terminal domain of Ant1 is processed to become the antirepressor Ant2, the expression modulator of the master repressor, C1 (5). The Rha protein is found in many temperate phages and interferes with infection of strains without an integration host factor (IHF) (6). Furthermore, Staley encodes a putative site-specific tyrosine recombinase with a conserved C-terminal catalytic domain of other phage integrases, such as P1 Cre and lambda Int (7).

### Nucleotide sequence accession number.

The genome sequence of phage Staley was contributed to GenBank under accession number KF669663.
